# Advances in Sustainable Polymeric Materials, 3rd Edition

**DOI:** 10.3390/polym18060729

**Published:** 2026-03-17

**Authors:** Cristina Cazan, Mihai Alin Pop

**Affiliations:** 1 Department of Product Design, Mechatronics and Environment, Transilvania University of Brasov, 500036 Brasov, Romania; 2 Department of Materials Science, Transilvania University of Brasov, 500036 Brasov, Romania

The increasing global demand for polymeric materials, combined with growing environmental concerns about fossil-based plastics, has significantly accelerated research into sustainable polymer systems. Conventional petroleum-derived polymers have contributed substantially to modern technological development, supporting applications in packaging, construction, transportation, electronics, and biomedical engineering. However, their extensive production and accumulation have resulted in serious environmental challenges, including greenhouse gas emissions, depletion of non-renewable resources, and persistent plastic pollution. These concerns have driven the development of sustainable polymeric materials designed to reduce environmental impacts while maintaining high performance and durability [1,2,3].

One key strategy in sustainable polymer science is the development of bio-based polymers derived from renewable resources, such as lignin, cellulose, starch, and other biomass feedstocks. These materials offer significant environmental advantages, including reduced carbon footprint, biodegradability, and lower dependence on fossil resources. In addition, advances in recycling technologies—including mechanical, chemical, and emerging biological recycling approaches—have enabled more efficient recovery and reuse of polymeric materials, supporting circular material flows and reducing waste accumulation [1,4,5]. Sustainable polymer composites incorporating industrial and agricultural waste have also emerged as promising alternatives to conventional materials, offering improved resource efficiency and reduced environmental impact while maintaining desirable mechanical and functional properties [6,7].

The circular economy concept has become a fundamental framework guiding the development of sustainable polymer systems. By promoting material reuse, recycling, and waste valorization, circular economy strategies can minimize environmental impact while maximizing resource efficiency. Waste-derived materials, including recycled plastics, industrial by-products, and biomass residues, can be successfully integrated into polymer matrices to produce high-performance composite materials suitable for engineering applications [5,7,8,9,10]. These approaches transform waste streams into valuable material resources, contributing to sustainable material management and reducing environmental pollution.

Sustainable polymeric materials have demonstrated significant potential across a wide range of advanced applications. Bio-based and recycled polymer systems are increasingly being utilized in construction, environmental remediation, biomedical engineering, and infrastructure development. Polymer composites reinforced with natural fibers or waste-derived fillers exhibit improved structural performance, durability, and environmental compatibility [11,12]. These developments highlight the important role of sustainable polymer science in addressing global environmental challenges and enabling the transition toward more resource-efficient material systems.

The rapid evolution of sustainable polymer science reflects its inherently multidisciplinary nature, integrating materials science, chemistry, environmental engineering, and industrial innovation. Advances in polymer synthesis, recycling technologies, and composite engineering have expanded the range of sustainable materials available for industrial and technological applications. As a result, sustainable polymer systems are widely recognized as essential components in achieving long-term environmental sustainability and supporting circular economic models [13].

One of the main research directions addressed in this Special Issue, “Advances in Sustainable Polymeric Materials, 3rd Edition”, is the development of bio-based polymers as viable alternatives to petroleum-derived materials. Stanley et al. [14] investigated the synthesis of high-molecular-weight poly(ethylene furanoate) (PEF), a fully bio-based polyester with strong potential to replace poly(ethylene terephthalate) in packaging applications. By optimizing solid-state polymerization parameters, the authors achieved significant improvements in molecular weight, crystallinity, and thermal stability, confirming the feasibility of producing high-performance renewable polymers suitable for industrial use. Complementing this work, Karimi et al. [15] presented a comprehensive review of inulin, a naturally occurring polysaccharide with a wide range of applications in pharmaceutical, biomedical, and industrial systems. The unique chemical structure, biodegradability, and functional versatility of inulin make it a promising sustainable biopolymer for advanced material applications.

Recent advances in bio-based functional polymer systems also include important developments in biomedical engineering and advanced manufacturing technologies. García-García et al. [16] investigated photo-crosslinkable hydrogels derived from natural polymers, including gelatin, alginate, and chitosan, demonstrating their excellent printability, mechanical stability, and biocompatibility for extrusion-based 3D printing applications. These hydrogels show strong potential for use in tissue engineering, regenerative medicine, and personalized biomedical devices. Similarly, Gök et al. [17] reported the sustainable production of bacterial cellulose using spent tea waste as a renewable carbon source. The resulting materials exhibited excellent mechanical properties, antimicrobial activity, and structural uniformity, confirming the feasibility of converting food waste into high-value, functional biomaterials suitable for biomedical, electronic, and environmental applications.

The valorization of agricultural and organic waste represents another important focus of this Special Issue. Agricultural residues and biomass waste offer abundant renewable resources for developing sustainable polymer composites. Wu et al. [18] demonstrated the successful utilization of corn stalk fibers recovered from cattle manure as reinforcement materials in friction composites. The biological pre-treatment and optimized silane modification improved fiber–matrix compatibility, reduced water absorption, and enhanced tribological performance. These results highlight the potential of agricultural waste fibers as sustainable reinforcement materials that can improve composite performance while simultaneously reducing environmental impacts.

In addition to structural applications, sustainable polymer materials have demonstrated significant potential in environmental remediation technologies. Mchich et al. [19] developed eco-friendly hydrogel beads derived from seashell waste and alginate for the removal of heavy metals from aqueous solutions. These materials exhibited excellent adsorption capacity, favourable kinetics, and good reusability, demonstrating their suitability for water purification applications. Similarly, Segneanu et al. [20] developed innovative nanocomposites based on waste-derived components, including eggshells, fly ash, magnetite nanoparticles, and biopolymers such as carrageenan and chitosan. The engineered materials achieved very high chromium removal efficiency, highlighting the synergistic effects of combining inorganic waste-derived materials with polymer matrices to develop advanced environmental remediation systems.

Further contributions focused on the removal of organic pollutants and the development of sustainable functional polymer systems. Duarte et al. [21] developed lignin-based rigid foams with excellent adsorption capacity for methylene blue, as well as flame-retardant and recyclable properties. The use of lignin, a widely available renewable aromatic biopolymer, represents an important step toward replacing fossil-based phenolic materials. In addition, Sriani et al. [22] demonstrated the successful upcycling of expanded polystyrene waste into functional membrane systems for microalgae harvesting. The developed membranes showed excellent permeability, structural stability, and biomass recovery efficiency, demonstrating the potential to convert plastic waste into valuable functional materials for environmental and industrial applications.

Sustainable polymer composites have also demonstrated their effectiveness in important applications in infrastructure and construction materials. Kim et al. [23] investigated the use of crushed bottom ash as a filler in polymer-modified asphalt mixtures. Their results demonstrated improved mechanical performance, moisture resistance, and durability, confirming the feasibility of incorporating industrial waste into high-performance infrastructure materials. Similarly, Ohm et al. [24] evaluated the use of crushed recycled marble stone powder as a sustainable filler in asphalt mixtures containing recycled tyre rubber. The incorporation of waste-derived fillers improved structural stability, deformation resistance, and mechanical performance, highlighting the effectiveness of circular economy approaches in infrastructure development.

Collectively, the studies in this Special Issue demonstrate significant progress in sustainable polymer science. Bio-based polymers derived from renewable resources have demonstrated strong potential to replace conventional petroleum-based materials while maintaining comparable or improved performance. The use of waste-derived materials in polymer composites represents an effective strategy for reducing environmental impact, improving resource efficiency, and supporting circular economic principles.

Furthermore, sustainable polymer systems have shown great potential for environmental remediation, biomedical engineering, advanced manufacturing, and infrastructure applications. These developments highlight the versatility and adaptability of polymer materials in addressing current environmental and technological challenges.

Despite these advances, several challenges remain in scaling laboratory-scale developments for industrial production. Future research should focus on improving material performance, optimizing processing methods, and evaluating long-term durability and lifecycle sustainability. Interdisciplinary collaboration among materials scientists, chemists, engineers, and environmental researchers will be essential to accelerating the development and implementation of sustainable polymer technologies.

In conclusion, the contributions presented in this Special Issue demonstrate the transformative potential of sustainable polymeric materials derived from renewable resources and waste streams. These advances support the transition toward more sustainable material systems, reduce environmental impact, and contribute to the development of circular economic solutions. Sustainable polymer science will continue to play a critical role in addressing global environmental challenges and enabling future technological innovation.
